# Effect of Biological Environment on Polyester Surgical Suture's Physical Properties: An Experimental Study in Rats

**DOI:** 10.7759/cureus.6303

**Published:** 2019-12-06

**Authors:** Dominykas Stankevicius, Justinas Jonusas, Violeta Zalgeviciene, Sigitas Ryliskis

**Affiliations:** 1 Department of Anatomy, Histology, and Anthropology, Vilnius University Faculty of Medicine, Vilnius, LTU; 2 Department of Radiology, Nuclear Medicine and Medical Physics, Vilnius University Faculty of Medicine, Vilnius, LTU; 3 Department of Anatomy, Histology and Anthropology, Vilnius University Faculty of Medicine, Vilnius, LTU; 4 Clinic of Rheumatology, Orthopaedics Traumatology and Reconstructive Surgery, Vilnius University Faculty of Medicine, Vilnius, LTU

**Keywords:** acromioclavicular dislocation, loss of reduction, partial failure, physical properties, polyester suture

## Abstract

Objective

The following study aims to analyze the alteration of nonabsorbable polyester surgical suture physical properties after in vivo incubation.

Methods

A comparative study of braided nonabsorbable polyester 2/0 (U.S.P) sutures was performed. The control in vitro group and three experimental in vivo subgroups, composed of ten sutures in each, were created. All 30 experimental sutures were implanted into a total of 15 laboratory rats subcutaneous tissue (two sutures in each rat) and removed after seven, eight and nine weeks, respectively. Further, they were attached to the mechanical testing device and affected with a mechanical force, increasing the load by 0,1 N/s until complete breakage. Tensile strength (TS), failure displacement (FD), failure strain (FS) and failure stress (FST) were measured at the point of failure and compared to the same parameters of the control group.

Results

No statistically significant difference was found in the physical parameters of the samples between the experimental and control groups (TS [*p* = 0.358], FD [*p* = 0.258], FS [*p* = 0.258] FST [*p* = 0.358]). A statistically significant difference was found in the failure load between sutures that break on the knot site (KS) and the rest of the samples: significantly less force was needed to break the suture on the KS. Moreover, most of the breaks on the KS occurred in sutures that were incubated for the longest period of nine weeks (*n* = 4). An anomaly of partial failure (PF) was noticed. Sutures with PF elongated significantly more compared to the sutures that did not undergo PF in the control and in experimental groups (*p* = 0,044; *p* = 0,017; *p* = 0,016; *p* = 0,013).

Conclusion

The biological environment had no radical aftereffects to the suture’s physical properties. In vivo exposure may cause the suture to break on the KS more frequently and may lead to PF, when a few sutures composed of fiber fail. Sutures that undergo PF tend to elongate further.

## Introduction

Surgical sutures nowadays are an inseparable part of any surgery. They not only play a huge role in wound healing but can also become an inflammation or allergy source. For this reason, any surgical suture must be non-reactive to tissues, maintain good knot security, and be strong and easily handled.

While there are various surgical techniques treating acromioclavicular (AC) joint dislocation, suture-button method or surgery with autograft tendon using nonabsorbable polyester suture remains widely performed and analyzed [1-3]. Another suture-loop technique uses polyester sutures as well [4-5]. One of the most common complications after AC joint stabilization surgery is the loss of reduction [6]. Recently published papers reveal the main risk factors such as wrong clavicle tunnel location, button migration, osteoporosis and suture breakage for this complication to occur [7-8]. We predict that* *an in vivo environment may affect polyester suture's material and cause its physical properties to become worse leading to loss of reduction. A few published works analyzed maximum load to failure, elongation and other physical properties of various surgical sutures [9-11]. One of the studies has shown that polypropylene sutures preserve their stability after being taken out of a living organism, while catgut and Caprosyn lose their tensile strength (TS) [12]. In another study, conducted by Daniel A. Muller and colleagues, it was noticed that absorbable monofilament, polydioxanone, and non-absorbable polyester sutures retain stable material properties after two months of incubation under physiological conditions [11]. However, the polyester suture’s alteration and expiry while sewn in a living organism are still not well known.

The aim of this study is to analyze the alteration of nonabsorbable polyester suture’s physical features such as TS, failure displacement (FD), failure strain (FS), and failure stress (FST) after being kept in vivo (rat) and observe its compliance in AC joint dislocation surgery. We speculate that these properties will worsen after in vivo incubation.

## Materials and methods

Creation of study groups

A comparative study of braided nonabsorbable polyester 2/0 (U.S.P; Ethibond Excel) sutures was performed in Open-access Centre of Biomedical Physics Laboratory, Nacional Cancer Institute, Vilnius, Lithuania. All sutures were purchased at the same time and were within their expiration date. Sutures were checked for imperfections before the experiment. Durability and TS tests were performed after* *in vivo and in vitroconditions.

Two groups were designed for the current study:

1. Control in vitro group (10 sutures were tested after extracting them from sterile packaging).

2. Experimental in vivo group was divided into three subgroups with 10 sutures in each, which were implanted into five rats (two sutures in each rat):

A. seven-weeks subgroup

B. eight-weeks subgroup

C. nine-weeks subgroup.

Implantation steps and technique

All experimental group sutures were implanted into Wistar clone rats and kept there for seven, eight and nine weeks accordingly. The experiment was designed in accordance with the requirements stated in the 2010/63/EU Directive and the Order of the Lithuanian State Food and Veterinary Service Director No B1-866; 31-12-2012. Approval of the Ethics Committee and permission for the experimentation was received from the State Food and Veterinary Service of Lithuania. Fifteen standard laboratory rats were used during experiments. The average weight of a rodent was 224,8 g (range between 200 and 251 g). All rats were kept in separate boxes with a separate air filtering system for every box thus preventing infection spreading among the rats. All rodents were kept under 12 hours day-night cycle at 20-21ºC and fed accordingly to their needs during the experiment. Ketamine (standard dose of 40 mg/kg intramuscular injection) was used for anesthesia during suture implantation. Surgical sites were prepared with 98% ethyl alcohol solution. Two 1-cm-wide cuts of skin were performed perpendicular to the spine with the first cut at the height of the first thoracic vertebra and second at the height of the first sacral vertebra. Canal, which connects both cuts, between hypoderm and muscle fascia was created, using mosquito forceps. Two sutures were implanted afterward through skin incisions in each rat subcutaneous tissue. Incisions were closed using two simple-interrupted stitches with 2/0 nonabsorbable sutures. After the procedure, rodents were injected with a single dose of cefazoline (rodent weight was considered when the concentration of antibiotic was calculated). No signs of inflammation, infection or sickness were recorded in any of the rats during the experiment. After the incubation time of seven, eight and nine weeks, sutures were removed from all rodents. It was done under aseptic conditions and intramuscular anesthesia using ketamine. Rodents were euthanized by intracarotid blood drawing later while still under the anesthesia.

Testing of physical parameters

TS was measured using Mecmesin MultiTest 2.5-i (2.5 kN) computer-controlled tensile and compression test system. For each tensile test, the suture was fixed on two hooks using “figure-eight” knots so ensuring a secure anchoring. Increasing load at the speed of 0.1 N/s was applied to the suture during the experiment. Other properties that were calculated were FS and FST. These parameters were calculated at a load of failure for all sutures. The length of all samples was calculated using a digital caliper and it was done before all measurements. Locations of suture ruptures were identified and documented. All measurements were done in units of the SI system. Further, all sutures were examined under the stereomicroscope and changes of the surface were captured.

Statistical analysis

During data analysis, the normality of all parameters was tested using Kolmogorov-Smirnov test. A Shapiro-Wilk test (*p* > 0.05) and inspection of data’s histograms, Q-Q and box plots revealed that all data were distributed non-normally. Non-parametric tests were used further. Non-parametric Kruskal-Wallis test was used to determine if there is any significance among the groups of sutures and physical parameters. When a statistically significant difference was observed, the non-parametric Mann-Whitney U test was used between the groups. The level of significance (*p*) was chosen to be less than 0.05. IBM SPSS Statistics 21 and MS Excel 2016 were used for the statistical analysis. Graphical data were analyzed using Origin 2018 data processing software.

## Results

Received data and calculations revealed that in vivo environment has no radical aftereffects to suture’s physical properties comparing control and experimental groups: TS (*p* = 0.358), FD (*p* = 0.258), FS (*p* = 0.258), FST (*p* = 0.358). However, the empirical analysis showed that there was a visible increase of failures on the knot site (KS) in experimental subgroups. While there were no failures on the KS in the control group, we did observe four failures on the KS in sutures that were kept in rodents for nine weeks (Table 1).

**Table 1:** Values of measured physical parameters at failure point Data are shown as means with a standard deviation KS, knot site; PF, partial failure

Suture group	Tensile strength (N)	Failure displacement (m)	Failure strain (Δm/l)	Failure stress (MPa)	Failure on the KS (No.)	PF of suture (No.)
Control group (n = 10)	134,4 ± 10,9	0,028 ± 0,002	16,8 ± 1,1	497,7 ± 40,6	0	2
7-week subgroup (n = 10)	141,1 ± 6,3	0,031 ± 0,003	18,4 ± 1,8	522,6 ± 23,3	1	3
8-week subgroup (n = 10)	140,5 ± 7,9	0,029 ± 0,004	18,2 ± 2,1	520,3 ± 29,4	2	5
9-week subgroup (n = 10)	140,7 ± 4,0	0,030 ± 0,004	18,1 ± 2,5	521,2 ± 14,8	4	6

Furthermore, statistical analysis revealed a correlation in failure load between the sutures that break on the KS and the rest of the samples. Significantly less force was needed to break the suture on the KS (Table 2).

**Table 2:** Comparison of failure load between sutures that failed on the knot site and the rest of suture Data are shown as means with a standard deviation. KS, knot site

Suture group	Failure load on the KS (N)	Failure load on the rest of suture (N)		Decrease of strength (%)	
					p-value
Control group (n = 10)	No failures recorded		134,4 ± 10,9 (n = 10)	NaN	NaN
7-weeks subgroup (n = 10)	126,9 (n = 1)		142,7 ± 4,1 (n = 9)	11.1	0,016
8-weeks subgroup (n = 10)	127,1 ± 5,4 (n = 2)		143,8 ± 3,6 (n = 8)	11.6	0,034
9-weeks subgroup (n = 10)	137,1 ± 1,6 (n = 4)		143,2 ± 3,1 (n = 6)	4.3	0,019

**Figure 1:**
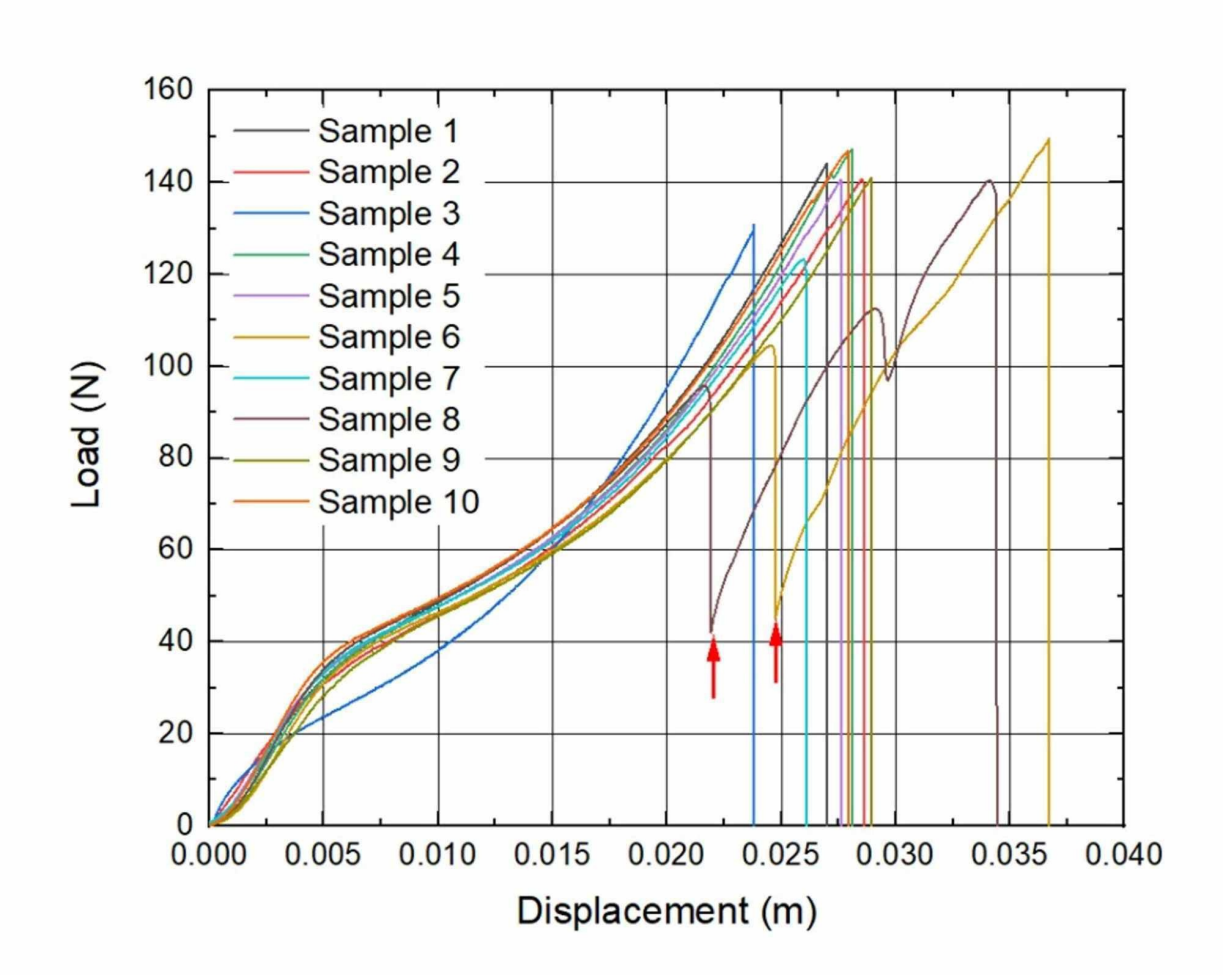
Load to displacement graphs of sutures: Control group Arrows mark the sudden loss of load force (PF). *n* = 2 PF, partial failure

**Figure 2:**
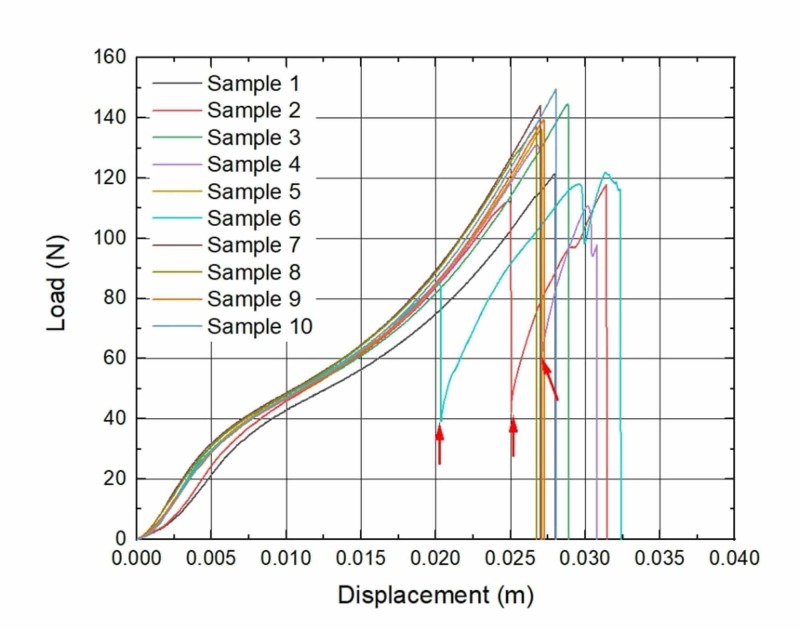
Load to displacement graphs of sutures: Seven-week implantation subgroup Arrows mark the sudden loss of load force (PF). *n* = 3 PF, partial failure

**Figure 3:**
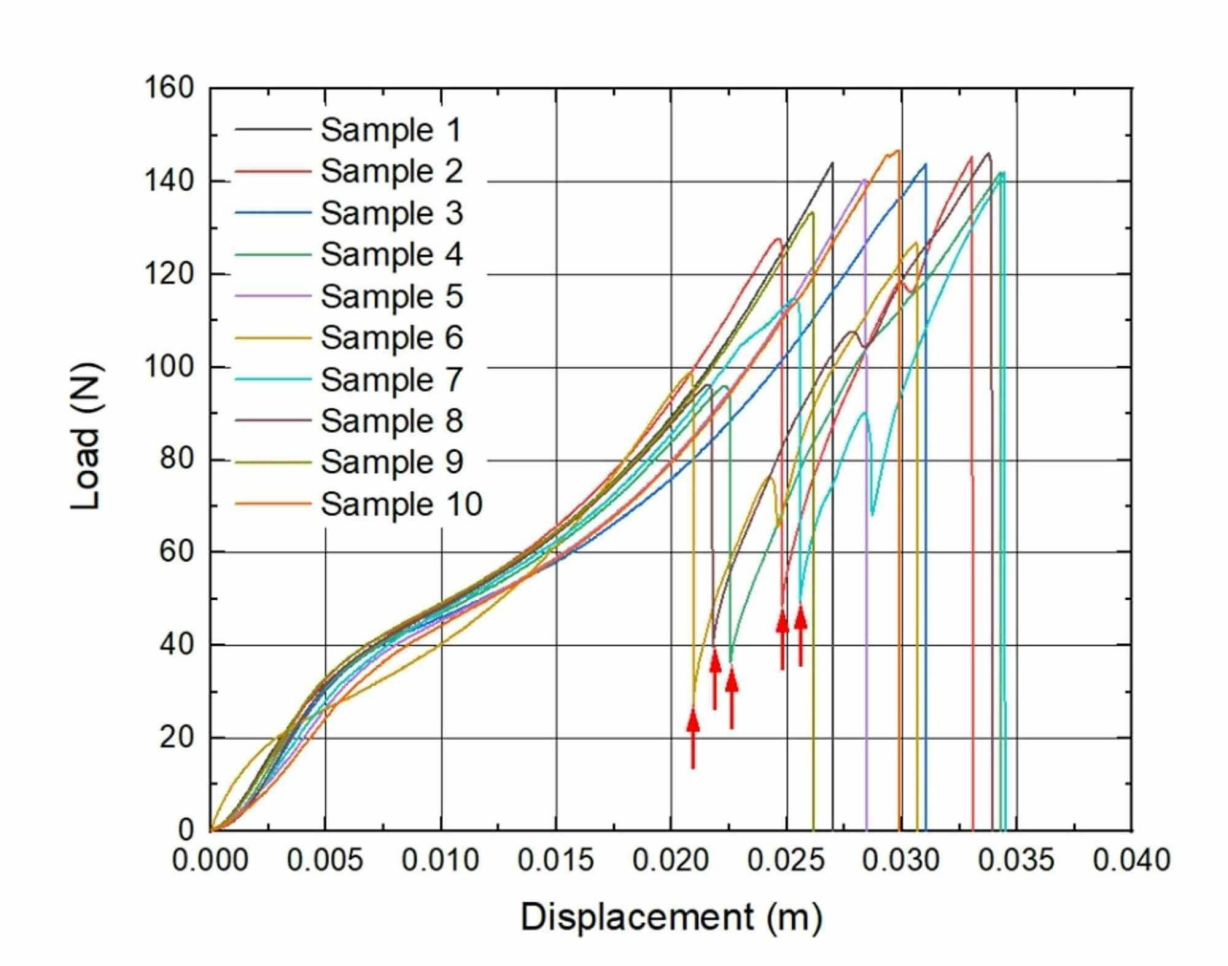
Load to displacement graphs of sutures: Eight-week implantation subgroup Arrows mark the sudden loss of load force (PF). *n* = 5 PF, partial failure

**Figure 4:**
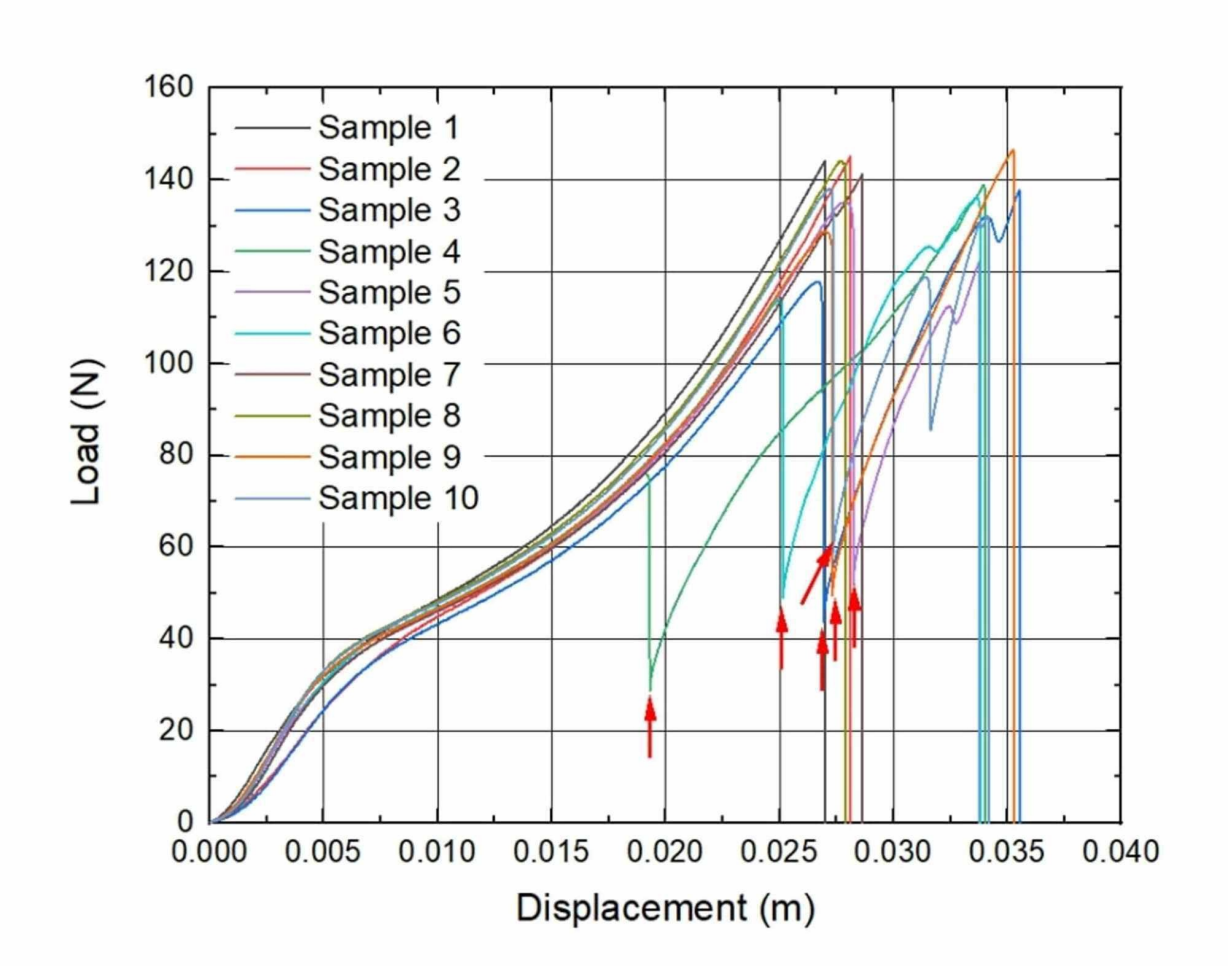
Load to displacement graphs of sutures: Nine-week implantation subgroup Arrows mark the sudden loss of load force (PF). *n* = 6 PF, partial failure

During the analysis of load-to-displacement graphs, an unexpected finding of partial failure (PF) was noticed: a sudden loss of tensile force was visible, but the suture did not totally break. In the seven-week subgroup, all three samples that have undergone PF were removed from different rats; in the eight-week subgroup, two samples were removed from the same rat and the other three from different ones; in the nine-week subgroup, two from the same rat and other four from different rats. Moreover, sutures with PF tended to expose higher displacement which is visible in graphs (Figures 1-4).

There was a significant displacement at failure load between sutures with PF and without PF in experimental and control groups. Sutures that undergo PF tended to elongate more significantly (Table 3).

**Table 3:** Comparison of displacement at failure load between sutures with PF and without PF Data are shown as means with a standard deviation. PF, partial failure

Suture group	Displacement of sutures with PF (m)	Displacement of sutures without PF (m)	p-value
Control group	0,035 ± 0,002 (n = 2)	0,027 ± 0,002 (n = 8)	0,044
7-weeks subgroup (n = 10)	0,031 ± 0,001 (n = 3)	0,027 ± 0,001 (n = 7)	0,017
8-weeks subgroup (n = 10)	0,033 ± 0,001 (n = 5)	0,028 ± 0,002 (n = 5)	0,016
9-weeks subgroup (n = 10)	0,034 ± 0,001 (n = 6)	0,027 ± 0,001 (n = 4)	0,013

**Figure 5:**
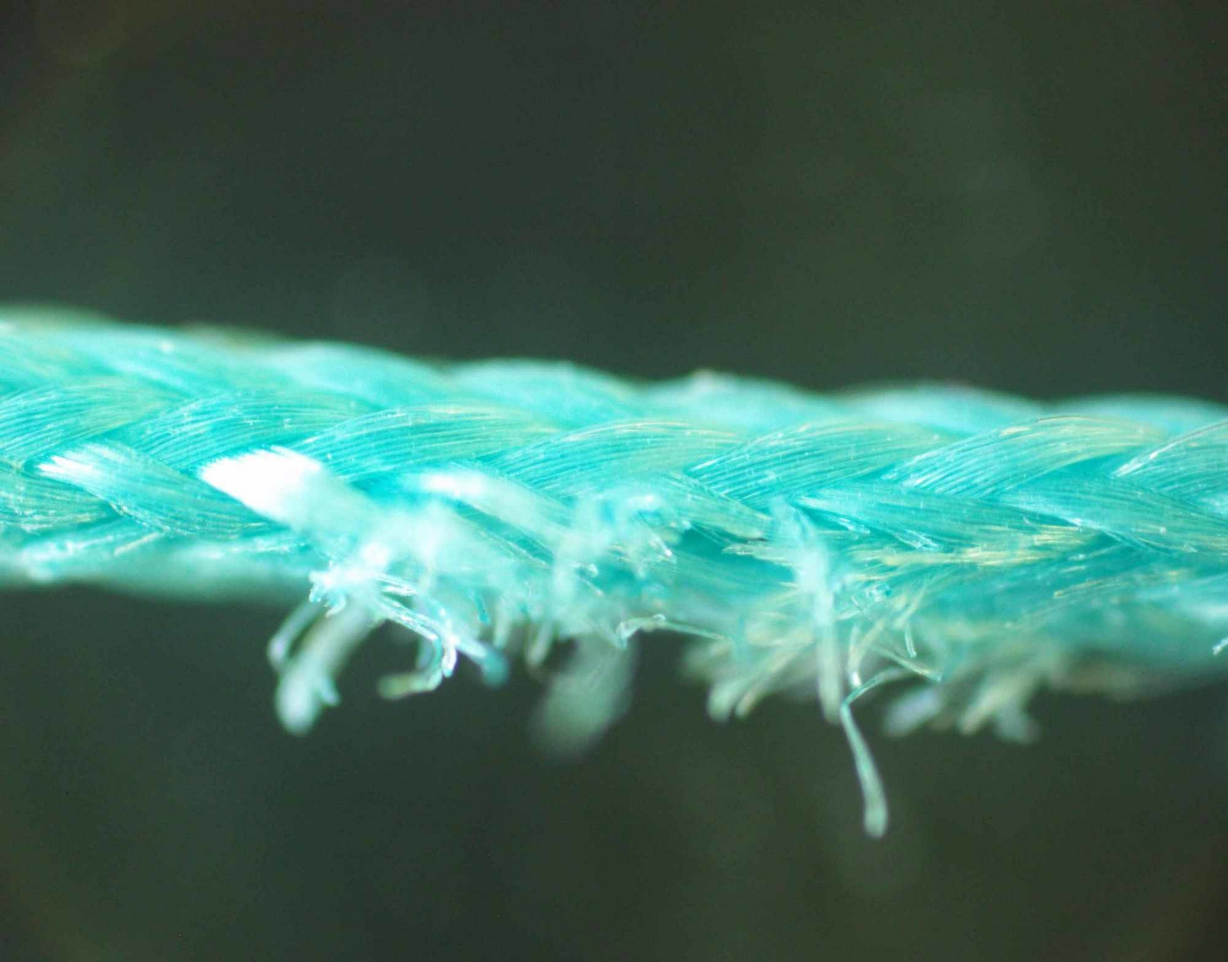
Microscopic inspection of suture deformation: Breakage of single fibers Magnification 40x

**Figure 6:**
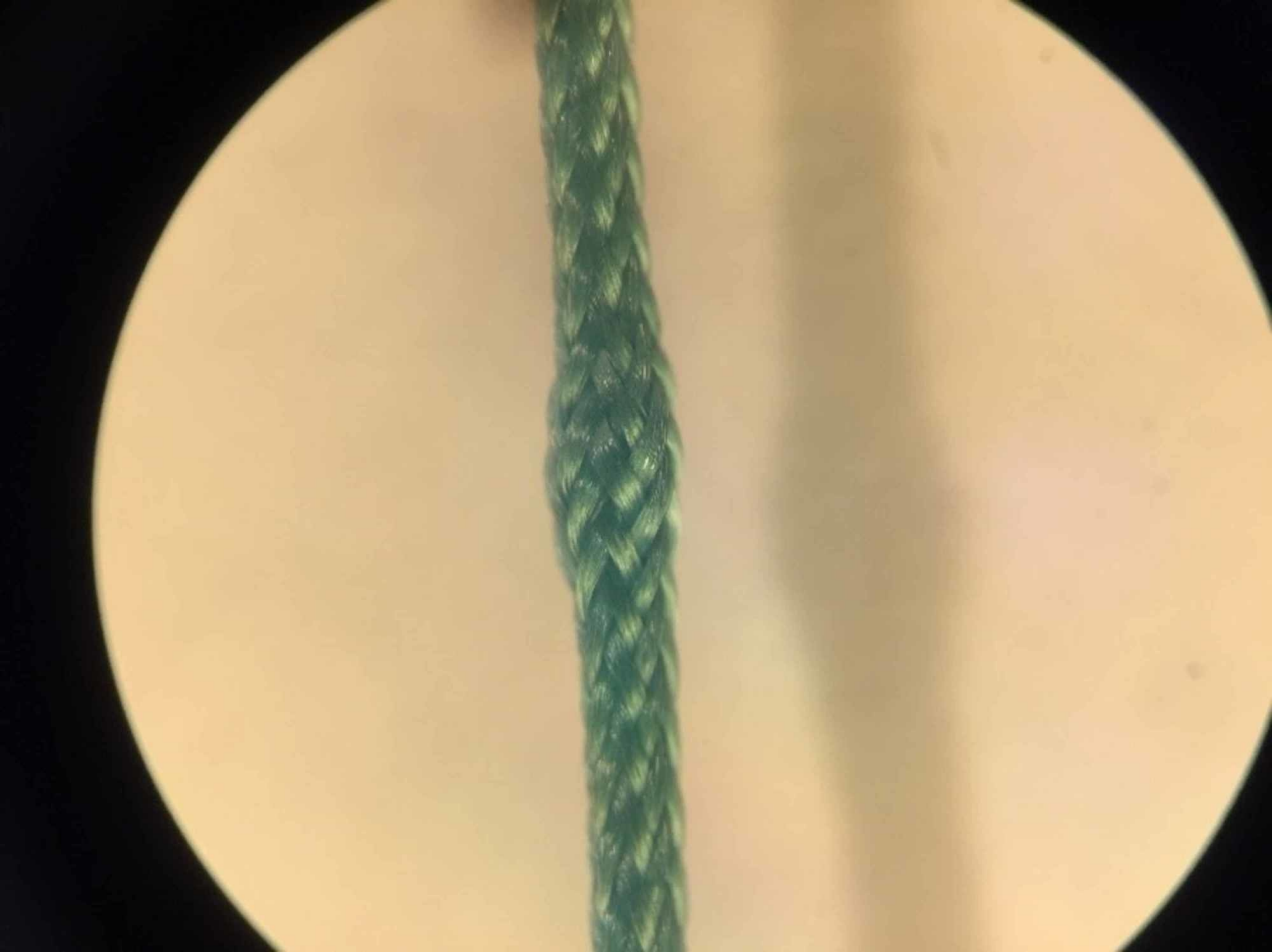
Microscopic inspection of suture deformation: Thickened area of sutures Magnification 28x

Sutures’ surface deformations such as single breakage of fibers and thickened areas were clearly seen during a microscopic inspection (Figures 5-6).

## Discussion

Even though sutures are an inseparable part of any surgeon’s work routine, there are only a few works analyzing how in vivo environment affects the suture material and its physical properties. The most important finding in this work is the observation of sutures PF after keeping them in rats.

The research conducted by Steven E. Naleway and coauthors showed that the KS dramatically decreases suture's TS [13]. Such a statement is confirmed by our findings. During the analysis of extracted sutures that were incubated in rodents, we did observe an increased rate of failures on the KS depending on their incubation time (Table 1). There were no failures on KS in the control group, one failure was recorded in the seven-weeks subgroup, two and four failures were recorded in eight- and nine-week subgroups, respectively. Moreover, significantly less load force was needed to break the suture on the KS (Table 2). This finding is relevant to the clinical use of sutures. It has been proved that after the coracoclavicular ligament reconstruction, one of the complications is loss of reduction [1,14]. Such loss of compression may result from the rupture of sutures on the KS [8,15]. Surgeons should take into consideration the fact that during the time in internal medium, sutures may lose their initial TS and take precautionary measures to avoid that.

A study of M.A. Tolga Muftuoglu and coauthors showed that nonabsorbable sutures lose their TS after in vivo incubation: Polypropylene and silk sutures lost their initial strength by 16% and 8%, respectively [16]. We did not observe any kind of significant loss of TS when the control group in vitro of brand-new sutures was compared with the experimental in vivo group sutures that were kept in rodents for seven, eight and nine weeks. During the investigation of force-displacement graphs, we observed anomalies in curves when a sudden loss of load force was visible, but suture did not totally break (Figures 1-4). We called these anomalies PF of sutures. We believe that PF occurs when a few sutures composed of fibers break. The failure of single fibers was clearly seen under the stereomicroscope (Figure 5). Moreover, we observed a significant displacement between the sutures with PF and sutures without PF in the experimental and control groups: sutures with PF displaced significantly more before breaking. This is also visible in the graph of the experimental study performed by Gomide et al., where the TS of the same sutures was measured at the rupture point [9]. It is important to emphasize that PF occurs more frequently within every week of implantation. It may be related to the incubation time which is needed for the biological environment to affect the sutures. These findings may have a link with compression loss within the first year after the correction of AC dislocation [17].

PF of the suture may be explained by the research of E. Karaca and A.S. Hockenberger where the breaking morphology of nonabsorbable polyester suture was researched [18]. Filaments tend to migrate toward the suture axis on loading and endure higher TS and displacement until total break during load. Changes in polyester suture surface after removal of load were described: Fibers rearrange across the diameter resulting in the thick and thin regions along the suture. Familiar deformation of the thickened area was observed during our microscopic examination of suture (Figure 6). PF phenomenon observed during this work might determine such changes in the suture’s surface and length.

The same paper tested two braided (polyester, silk) and two monofilament (polyamide, polypropylene) sutures (0, 2/0, 3/0 U.S.P) [18]. Silk and polyamide sutures showed a significant decrease in their TS while polypropylene and polyester sutures retained their TS after three and eight weeks under in vivoconditions. Moreover, a considerable increase of TS was recorded in the group of polyester 3/0 USP sutures. Familiar outcomes of the polyester suture were seen in our job - suture’s TS increased after in vivo incubation. Such a phenomenon may be explained by the PF mechanism which might be more common in thinner polyester sutures. Further investigation is needed to confirm it.

## Conclusions

The biological environment has no radical aftereffects to the physical properties of braided polyester suture. However, it may lead to PF and determine more common suture breakage on the KS. Sutures that undergo PF tend to elongate more and might cause the loss of reduction after AC joint dislocation surgery.
